# Impaired Inflammatory Responses in Murine Lrrk2-Knockdown Brain Microglia

**DOI:** 10.1371/journal.pone.0034693

**Published:** 2012-04-09

**Authors:** Beomsue Kim, Myung-Soon Yang, Dongjoo Choi, Jong-Hyeon Kim, Hye-Sun Kim, Wongi Seol, Sangdun Choi, Ilo Jou, Eun-Young Kim, Eun-hye Joe

**Affiliations:** 1 Department of Pharmacology, Ajou University School of Medicine, Suwon, Korea; 2 Neuroscience Graduate Program, Ajou University School of Medicine, Suwon, Korea; 3 InAm Neuroscience Research Center, Wonkwang University, Sanbon Hospital, Gunpo, Korea; 4 Department of Molecular Science and Technology, Ajou University School of Medicine, Suwon, Korea; 5 Chronic Inflammatory Disease Research Center, Ajou University School of Medicine, Suwon, Korea; 6 Institute for Medical Sciences, Ajou University School of Medicine, Suwon, Korea; National Institute of Health, United States of America

## Abstract

LRRK2, a Parkinson's disease associated gene, is highly expressed in microglia in addition to neurons; however, its function in microglia has not been evaluated. Using Lrrk2 knockdown (Lrrk2-KD) murine microglia prepared by lentiviral-mediated transfer of Lrrk2-specific small inhibitory hairpin RNA (shRNA), we found that Lrrk2 deficiency attenuated lipopolysaccharide (LPS)-induced mRNA and/or protein expression of inducible nitric oxide synthase, TNF-α, IL-1β and IL-6. LPS-induced phosphorylation of p38 mitogen-activated protein kinase and stimulation of NF-κB-responsive luciferase reporter activity was also decreased in Lrrk2-KD cells. Interestingly, the decrease in NF-κB transcriptional activity measured by luciferase assays appeared to reflect increased binding of the inhibitory NF-κB homodimer, p50/p50, to DNA. In LPS-responsive HEK293T cells, overexpression of the human LRRK2 pathologic, kinase-active mutant G2019S increased basal and LPS-induced levels of phosphorylated p38 and JNK, whereas wild-type and other pathologic (R1441C and G2385R) or artificial kinase-dead (D1994A) LRRK2 mutants either enhanced or did not change basal and LPS-induced p38 and JNK phosphorylation levels. However, wild-type LRRK2 and all LRRK2 mutant variants equally enhanced NF-κB transcriptional activity. Taken together, these results suggest that LRRK2 is a positive regulator of inflammation in murine microglia, and LRRK2 mutations may alter the microenvironment of the brain to favor neuroinflammation.

## Introduction

Parkinson's disease (PD), the second-most common neurodegenerative disorder, is caused by degeneration of dopaminergic neurons in the substantia nigra (SN). Leucine-rich repeat kinase 2 (LRRK2), an autosomal dominant gene in familial PD, consists of six functional domains: ankyrin-like (ANK), leucine-rich repeat (LRR), Ras of complex (ROC), C-terminal of ROC (COR), kinase, and WD40 domains. To date, 51 disease-associated mutations in LRRK2 have been identified in familial or sporadic cases. These mutations are scattered throughout the entire LRRK2 gene, and include R1441C/G/H in the ROC domain, G2019S in the kinase domain, and G2385R in the WD40 domain [1]–[4]. Although there is evidence showing that expression of pathological LRRK2 mutations is sufficient to cause neurotoxicity *in vitro* [5], [6], transgenic LRRK2 mutant mice show little or no obvious degeneration of dopaminergic neurons [7]–[10]. Therefore, it has been suggested that certain changes in the brain microenvironment may cooperate with genetic defects to promote the development of PD [11].

Although the etiology of PD is poorly understood, it is generally accepted that inflammatory processes are a risk factor [12], [13]. It has been reported that activated microglia expressing a large number of inflammatory genes are elevated in the striatum and SN region of PD patients and in animal models of PD, and mediate dopaminergic neuronal death [12], [14], [15]. Immune cells, such as B lymphocytes, lymphoblasts, monocytes, and microglia, express high levels of LRRK2, suggesting a role in immune responses [16]–[18]. Blood mononuclear cells from patents bearing an LRRK2-G2019S mutation exhibits large differences in transcriptional profiles of immune-response genes compared to healthy control cells [19]. Moreover, genome-wide association studies have identified single-nucleotide polymorphisms (SNPs) of LRRK2 in a susceptibility locus for chronic inflammatory diseases such as Crohn's disease and leprosy [20], [21]. However, few details about the roles of LRRK2 in immune response are known.

In this study, we demonstrate that LRRK2 acts through regulation of p38 MAPK and NF-κB signaling pathways to stimulate microglial inflammatory responses. These results suggest that LRRK2 mutations could change the microenvironment of brain, and thereby trigger and/or enhance the pathogenesis of PD.

## Methods

### Reagents

DMEM and FBS were from Hyclone (Logan, UT, USA). AMV reverse transcriptase was from Genedepot (Barker, TX, USA). PCR primers and biotin-labeled DNA probe were obtained from IDT (Coralville, IA, USA). The expression vectors pCDNA3-hCD14, pFlag-CMV1-hMD2, and pCDNA3-hTLR4-YFP were obtained from Addgene (Cambridge, MA, USA). 3× myc-tagged human LRRK2-WT, LRRK2-R1441C, LRRK2-D1994A, and LRRK2-G2019S expression plasmids were prepared by cloning into pcDNA3.1 as described previously [22], [23]. Antibodies to mouse inducible nitric oxide synthase (iNOS) were purchased from Millipore (Temecula, CA, USA); antibodies to mouse Lrrk2, human LRRK2 (hLRRK2), phosphorylated (p)-p38 (Thr180/Tyr182), p-JNK (Thr183/Tyr185), p-ERK (Thr202/Tyr204), and p-MKK3/6 (Ser189/207) were from Cell Signaling Technology (Danvers, MA, USA). Antibodies to Myc were from Sigma (St. Louis, MO, USA); antibodies to α-tubulin, p38, MKK3/6, NF-κB p65, and NF-κB p105/p50 were from Santa Cruz Biotechnology (Santa Cruz, CA, USA). Lipoteichoic acid (LTA), CL097, and ODN 1668, were obtained from Invivogen (San Diego, CA, USA). Polybrene, puromycin, and other unspecified reagents were from Sigma.

### BV-2 microglia culture and construction of Lrrk2-knockdown microglia

BV-2, immortalized murine microglial cell line, were obtained from Dr. E.J. Choi (Korea University, Korea) and cultured as described previously [24]. BV-2 cells were originally generated by infecting primary microglia with a v-raf/v-myc recombinant retrovirus, and retained most of the properties of freshly isolated microglia [25]. Cells were subcultured at 70–80% confluence almost daily. For construction of Lrrk2-knockdown (Lrrk2-KD) microglia, cells were infected with lentiviral particles carrying three small inhibitory hairpin RNAs (shRNAs) specific for mouse Lrrk2 (shLrrk2, sc-45750-V, Santa Cruz Biotechnology). Lentiviral particles with non-targeted shRNAs (shNT, sc-108080, Santa Cruz Biotechnology) were used as a negative control. Cells (5×10^4^ cells/mL) were seeded in 12-well plates and incubated with fresh media containing polybrene (5 µg/mL) and lentiviral particles (1.0×10^5^ IFU) for 12 h. Infected cells were selected with puromycin (5 µg/mL for 2 d) and plated into 96-well plates at a density of 0.5 cells/well for cloning. After clonal selection with puromycin, seven Lrrk2-KD clones that expressed less than 20% of basal levels of Lrrk2 mRNA and four control clones that expressed normal levels of Lrrk2 mRNA were obtained.

### Quantitative real-time reverse transcription-polymerase chain reaction

Total RNA was isolated from microglia or whole bodies of flies using easy-BLUE reagent (iNtRON Biotechnology, Seoul, Korea). cDNA was prepared from total RNA using M-MLV reverse transcriptase and oligo d(T)_15_ primers, according to the manufacturer's instructions (Elpis Bio, Daejeon, Korea). cDNA and forward/reverse primers (200 nM) were mixed with KAPA SYBR Fast Master Mix (Cape Town, South Africa), and quantitative real-time reverse transcription-polymerase chain reaction (qRT-PCR) was carried out using an RG-6000 real-time amplification instrument (Corbett Research, Sydney, Australia) under the following conditions: denaturation at 95°C for 30 s, followed by 40 cycles of 95°C for 3 s and 60°C for 30 s. Threshold cycle (C_T_) values were calculated for each gene, and normalized to that of glyceraldehyde 3-phosphate dehydrogenase (Gapdh), used as an internal reference. Expression levels relative to controls, expressed as fold induction, were determined using the comparative C_T_ (2^−ΔΔCT^) method [26]. Details of primer sequences, product sizes, and melting temperatures (T_m_) are listed in Table 1. hLRRK2 was also analyzed by conventional RT-PCR.

**Table 1:** List of primer sequences used for qRT-PCR.^1^

Species	Gene	GeneBank ID	Size (bp)	Sequence (5′-3′)
Mouse	GAPDH	NM_008084.2	142	**Fwd-** GCCTTCCGTGTTCCTACC
				**Rev-** CCTCAGTGTAGCCCAAGATG
	LRRK2	NM_025730.3	145	**Fwd-** GCCACGAATCTCAATAGCAAG
				**Rev-** CCAAAGCCAAGCACAGTATTC
	iNOS	NM_010927.3	150	**Fwd-** GCAAACATCACATTCAGATCCC
				**Rev-** TCAGCCTCATGGTAAACACG
	TNF-α	NM_013693.2	134	**Fwd-** CTTCTGTCTACTGAACTTCGGG
				**Rev-** CAGGCTTGTCACTCGAATTTTG
	IL-1β	NM_008361.3	148	**Fwd-** TCCTGTGTAATGAAAGACGGC
				**Rev-** ACTCCACTTTGCTCTTGACTTC
	IL-6	NM_031168.1	614	**Fwd-** CAAGAGACTTCCATCCAGTTGC
				**Rev-** TTGCCGAGTAGATCTCAAAGTGAC

1 **Fwd**, Forward primer; **Rev**, Reverse primer.

### Western blot analysis

Cells were washed with PBS and lysed in modified RIPA buffer (10 mM Na_2_HPO_4_ pH 7.2, 150 mM NaCl, 1% Nonidet P-40 [NP-40], 0.5% Na-deoxycholate) containing protease inhibitors (2 mM PMSF, 10 µg/mL leupeptin, 10 µg/mL pepstatin, 2 mM EDTA). Total proteins (50–100 µg) were separated by SDS-PAGE, and transferred to nitrocellulose membranes. Membranes were incubated with primary antibodies, followed by incubation with horseradish peroxidase-conjugated secondary antibodies. Protein bands were visualized using an enhanced chemiluminescence system.

### ELISA

Culture media were collected, and the levels of mouse TNF-α and IL-6 were measured using an ELISA kit (Invitrogen, Carlsbad, CA, USA) according to instructions provided by Invitrogen.

### Measurement of nitric oxide

The amount of nitrite formed from nitric oxide (NO) was measured by mixing culture medium (50 µL) with an equal volume of Griess reagent (0.1% naphthylethylene diamine, 1% sulfanilamide, 2.5% H_3_PO_4_), and then measuring optical density at 540 nm [27].

### EMSA and supershift assays

NF-κB DNA-binding activity was measured using an EMSA, as described previously. BV-2 or TCM-HEK cells (3×10^6^ cells) were harvested and suspended in 900 µL hypotonic solution (10 mM HEPES pH 7.9, 10 mM KCl, 0.1 mM EDTA, 0.1 mM EGTA, 1 mM DTT, 0.5 mM PMSF) for 15 min, followed by incubation in hypotonic solution containing 0.5% (v/v) NP-40 for 5 min. Cells were then centrifuged at 500× g for 10 min at 4°C, and the pellet containing the nuclear fraction was resuspended in a solution containing 20 mM HEPES (pH 7.9), 20% (v/v) glycerol, 0.4 M NaCl, 1 mM EDTA, 1 mM EGTA, 1 mM DTT, and 1 mM PMSF. Samples were centrifuged at 10,000× g for 10 min, and supernatants (crude nuclear fractions) were collected and stored at −70°C until use.

Oligonucleotides for the NF-κB consensus binding sequence, 5′-AGT TGA GGG GAC TTT CCC AGG C-3′ (Santa Cruz Biotechnology), were end-labeled using T4 polynucleotide kinase (Promega, Madison, WI, USA) and [γ-^32^P] ATP (Perkin Elmer, Waltham, MA, USA). The labeled DNA probe (0.5 ng) was incubated for 30 min with 5 µg nuclear proteins in a reaction mixture containing 21.4 mM EDTA, 21.4 mM EGTA, 20% (v/v) glycerol, 0.29 mM ZnSO_4_, 10 ng/mL poly(dI-dC), 1 mM DTT, 0.4 mg/mL bovine serum albumin, and 8 mM MgCl_2_. For supershift assays, nuclear extracts were preincubated with 1 µg of a combination of anti-p65 and anti-p105/p50 antibodies for 10 min at 4°C before adding probe. Reaction mixtures were resolved by electrophoresis on 6% (w/v) polyacrylamide gels.

### DNA affinity precipitation assay

DNA affinity precipitation assays were performed as described previously, with minor modifications [28]. Briefly, 100 µg of nuclear protein extracts prepared as described for EMSAs was incubated with 5′-biotinylated DNA probe (25 nM) containing the NF-κB consensus motif, 5′-AGT TGA GGG GAC TTT CCC AGG C-3′, at 4°C for 1 h in DNA-binding buffer (20 mM Tris-Cl pH7.4, 1 mM EDTA, 0.1% Triton X-100, 1 µg poly(dI-dC), 1 mM DTT, 4% glycerol). The NF-κB probe–protein complexes were immobilized by incubating with streptavidin agarose resin (Invitrogen) at 4°C for 30 min with gentle rotation, centrifuged, and washed repeatedly with binding buffer. Collected protein complexes were separated by SDS-PAGE, and the NF-κB subunits bound to DNA probes were detected by immunoblotting with p65 and p50 antibodies. The levels of nuclear translocated NF-κB subunits were detected by resolving 10 µg of nuclear protein that had not been reacted with probe on SDS-PAGE gels, and then probing with antibodies against p65, p50, and TATA-binding protein (TBP, nuclear internal control) by Western blotting, as described above.

### Plasmid constructs

LRRK2-G2385R, a pathogenic hLRRK2 mutant containing a substitution in the WD domain, was constructed by site-direct mutagenesis. Briefly, a 3.8-kb *Xba*I fragment from a pcDNA3.1 construct containing the C-terminus of LRRK2-WT was subcloned into pSP72 (Promega). The LRRK2-G2385R mutation was introduced into the pSP72-hLRRK2-WT plasmid using a Quick Change site-directed mutagenesis kit (Stratagene, La Jolla, CA, USA) and the primers pG2385R-F (5′-ACT GAA AAA CTC TGT AGA CTA ATA GAC TGC GTG-3′) and pG2385R-R (5′-CAC GCA GTC TAT TAG TCT ACA GAG TTT TTC AGT-3′). The *Xba*I fragment from pSP72-LRRK2-G2385R was re-introduced into the *Xba*I-digested pcDNA3.1-hLRRK2-WT vector. The integrity and direction of the constructed clone were confirmed by sequencing.

### Luciferase reporter assay

BV-2 cells (6×10^5^ cells) plated in a 35-mm dish were transfected with 1.2 µg of a 5× NF-κB-luciferase reporter construct (Stratagene) and 0.3 µg CMV-β-gal (for normalization of transfection efficiency) using 3 µL of JetPEI (Polyplus Transfection, Illkirch, France). After 24 h, cells were treated with 100 ng/mL LPS for 3 h. Cells were lysed in passive lysis buffer (Promega). Firefly luciferase (Luciferase Assay System) and β-galactosidase (Beta-GLO Assay System) activities were measured according to the manufacturer's instructions (Promega).

Human embryonic kidney (HEK293T) cells were gifted from Dr. H. Suh-Kim (Ajou University, Korea) and maintained in high-glucose DMEM containing 10% FBS, 4 mM L-glutamine, and 1% penicillin/streptomycin. Cells were allowed to grow to approximately 80–90% confluence before sub-culturing. HEK293T cells were made LPS-responsive by seeding onto 24-well plates at a density of 1×10^5^ cells/well, and then co-transfecting with pCDNA3-hCD14, pFlag-CMV1-hMD2, and pCDNA3-hTLR4-YFP (50 ng each). For experiments, LPS-responsive HEK293T cells were co-transfected with the NF-κB-luciferase reporter (100 ng/well), pRL-Tk (5 ng/well), and 250 ng/well LRRK2 expression vector or empty pcDNA3 (mock) using 1 µL of JetPEI. After transfecting for 4 h, cells were washed with fresh culture medium, maintained for 20 h, and then treated with LPS for 6 h. Cells were lysed, and luciferase activity was measured using the Dual-Luciferase Assay System (Promega).

### Statistical analysis

Data were analyzed by Student's t-test or one-way ANOVA followed by post-hoc comparisons (Student-Newman-Keuls approach), using the Statistical Package for Social Sciences version 8.0 (SPSS Inc., Chicago, IL).

## Results

### Lrrk2 knockdown attenuates LPS-induced inflammatory responses in microglia

**Figure 1:**
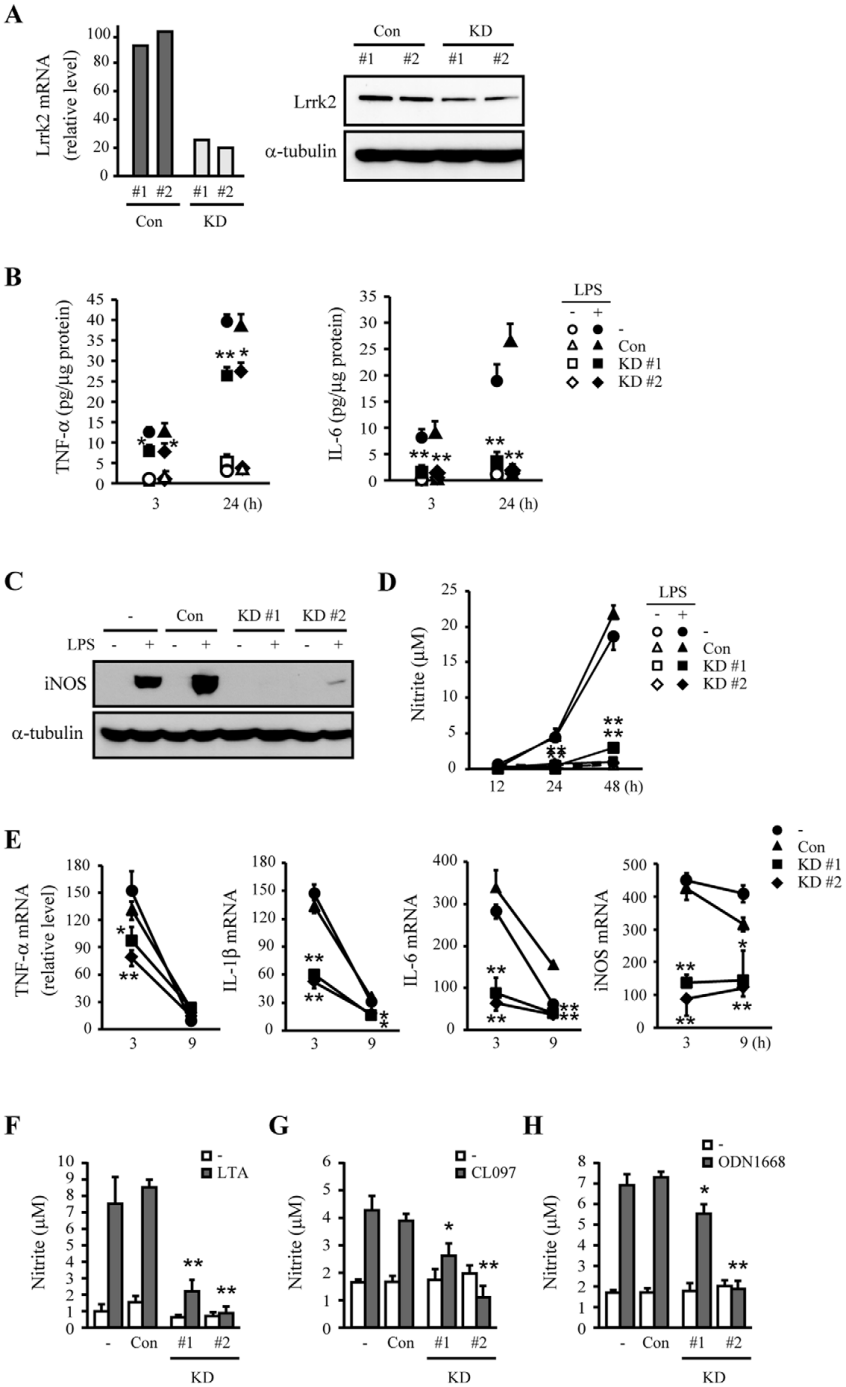
Attenuation of inflammatory responses in Lrrk2-KD microglia. (**A**) BV-2 microglia were infected with lentivirus expressing non-targeted (Con) or Lrrk2-targeted shRNA (KD). Two stable clones of each group were selected. Expression levels of Lrrk2 mRNA and protein were analyzed by qRT-PCR (left) and Western blotting (right), respectively. Parental (−), con, and KD cells were treated with or without 100 ng/mL LPS (B–E), 10 µg/mL LTA (F), 500 ng/mL CL097 (G), or 500 ng/ml ODN1668 (H) for indicated times (B, D, E), 12 h (C), 24 h (F), or 48 h (G, H). (**B**) TNF-α and IL-6 secretion into the culture medium were analyzed by ELISA. (**C, D, F–H**) iNOS protein expression was assayed by Western blotting (C), and NO release was measured using the Griess reagent, as described in Materials and Methods (D, F–H). (**E**) TNF-α, IL-1β, IL-6 and iNOS mRNA levels were analyzed by qRT-PCR. Gapdh mRNA and α-tubulin protein levels were analyzed as internal controls for qRT-PCR and Western blotting, respectively. Values are means ± SEMs (*p<0.05, **p<0.01 vs. control). Data are representative of at least three independent experiments unless indicated otherwise.

As a first step in examining the role of Lrrk2 in microglia, we developed stable murine Lrrk2 knockdown (Lrrk2-KD) microglia. For this, we infected BV-2 microglia with lentiviral particles encoding shRNAs targeting Lrrk2 or a non-targeting control shRNA. We chose two Lrrk2-KD clones and two control clones for these experiments. Lrrk2 mRNA and proteins levels were dramatically reduced in Lrrk2-KD microglia (Fig. 1A). We then analyzed the expression of the proinflammatory mediators TNF-α, IL-1β, and iNOS following LPS treatment. In Lrrk2-KD clones, TNF-α and IL-6 secretion was significantly decreased at 3 and 24 hours compared to untransfected parental (−) cells and control clones (Fig. 1B). iNOS protein expression and NO production (detected as NO-derived nitrite by 48 hours after activation) were also significantly reduced in Lrrk2-KD clones at 24 and 48 hours (Fig. 1C, 1D). Consistent with the results, a qRT-PCR analysis showed that LPS (100 ng/mL)-induced expression of IL-1β, IL-6, and iNOS mRNA was dramatically reduced in Lrrk2-KD clones at 3 and 9 hours (Fig. 1E). A significant decrease in TNF-α mRNA expression was only detected at 3 hours since its expression reduced to the basal level at 9 hours (Fig. 1E). We further examined whether Lrrk2 affects other TLR receptors that have potentials to induce inflammatory response in BV-2 microglia [29]. Using agonists for TLR2 (lipoteichoic acid, LTA, 10 µg/ml), TLR-7/8 (CL097, 500 ng/ml), and TLR-9 (ODN1668, 500 ng/ml), we found that Lrrk2-KD could regulate inflammatory responses mediated by these TLRs since nitric oxide production induced by these TLR agonists were significantly reduced in Lrrk2 KD clones (Fig. 1F–H). Similar results were obtained in *Drosophila* LRRK (dLRRK) loss-of-function mutant, LRRK^P1^ [30]: mRNA expression of antimicrobial proteins, attacin A and diptericin, was significantly reduced in LRRK^P1^ compared to wild type *Drosophila* at 5-, or 30-days (Figure S1). Taken together, these results indicate that LRRK2 is a positive regulator of inflammation.

### Lrrk2 knockdown specifically attenuates LPS-induced activation of p38 MAPK, but not JNK or ERK

**Figure 2:**
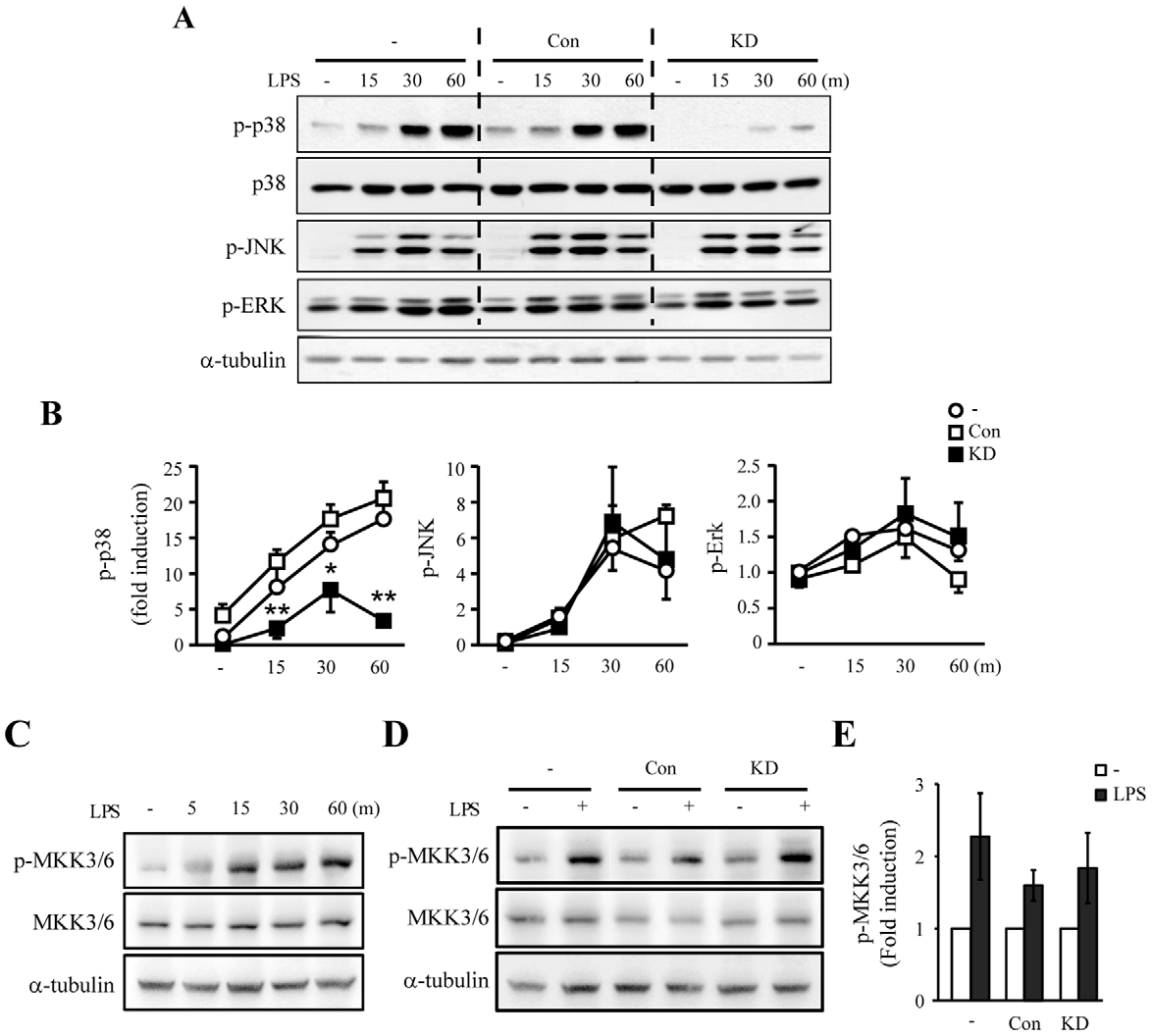
LPS-induced p38 phosphorylation is specifically inhibited in Lrrk2-KD cells. (**A, C, D**) Cells were incubated with LPS (100 ng/mL) for the indicated times (A, C) or 30 min (D), and the levels of phosphorylated p38 (p-p38), JNK (p-JNK), ERK (p-ERK), and total p38 (A) or total and phosphorylated MKK3/6 (C, D) were determined by Western blotting. α-tubulin was used as an internal control. (**B, E**) Band intensities of p-p38 (B) and p-MKK3/6 (D) were quantified using a densitometer. Values are means ± SEMs of three independent experiments (*p<0.05, **, *p*<0.01 vs. control). Data are representative of three independent experiments.

MAPKs are critical signaling molecules in LPS-induced microglial inflammatory processes [31]. Therefore, we examined whether activation of MAPKs was impaired in Lrrk2-KD microglia by analyzing phosphorylation of p38, JNK, and ERK following LPS stimulation. Parental cells and control cells showed similar levels of phosphorylated MAPKs (Fig. 2A, 2B). Interestingly, the levels of phosphorylated p38 (p-p38) were specifically attenuated in Lrrk2-KD cells between 15 and 60 min after LPS treatment compared to that in parental and control cells while the levels of p-ERK and p-JNK were not different (Fig. 2A, 2B). The other Lrrk2-KD clone also showed attenuated p-p38 levels in response to LPS (data not shown). Next, we analyzed activation of MKK3/6, an upstream kinase of p38 MAPK. In response to LPS, phosphorylation levels of serine 189/207 of MKK3/6 (p-MKK3/6) increased from 15 to 60 min (Fig. 2C). However, p-MKK3/6 levels were not significantly different in parental, control, and Lrrk2-KD cells (Fig. 2D, 2E).

### Lrrk2 knockdown attenuates LPS-induced activation of NF-κB and increases the DNA-binding activity of the NF-κB p50 subunit

**Figure 3:**
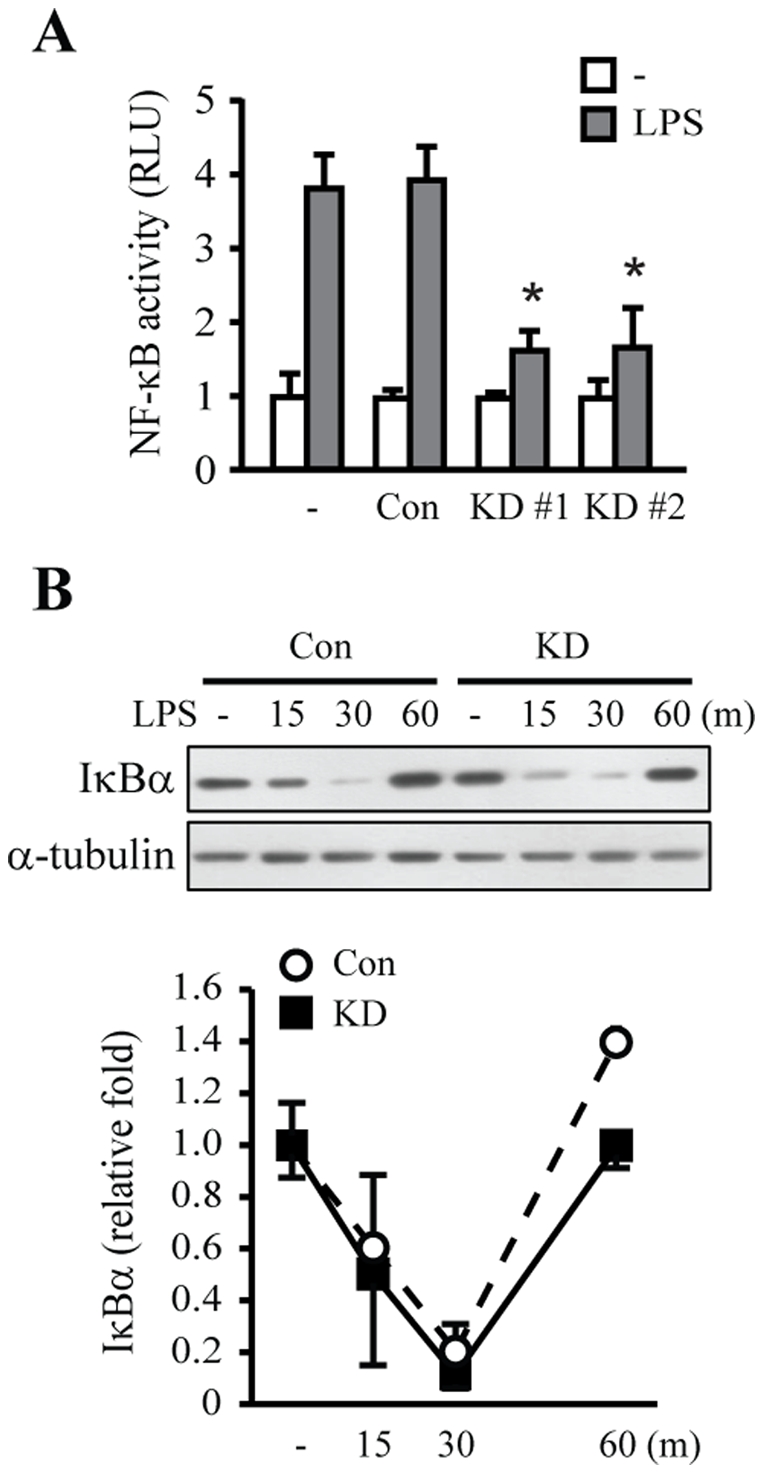
NF-κB transcriptional activity, but not IκB degradation, is decreased in Lrrk2-KD cells. (**A**) Parental (−), control (Con) and Lrrk2-KD (KD) cells were transfected with a 5× NF-κB-luciferase reporter plasmid (pDNA), and luciferase activity was measured 3 h after LPS stimulation. (**B**) Cells were treated with LPS for the indicated times, and IκB levels were analyzed by Western blotting. Relative IκB levels were quantified using α-tubulin as an internal control. Values in (A) and (B) are means ± SEMs of three independent experiments (*p<0.05).

The transcription factor NF-κB is important in mediating the expression of inflammatory genes in microglia [32]–[34]. To assess the function of Lrrk2 in the process of inflammatory activation, we analyzed the transcriptional activity of NF-κB in Lrrk2-KD microglia by measuring the activity of a luciferase reporter expressed under the control of a promoter containing five repeats of an NF-κB-binding sequence. LPS (100 ng/mL) increased luciferase activity by 3–4 fold in parental (−) and control cells; in contrast, luciferase activity was increased only 1–2 folds in both of Lrrk2-KD clones (Fig. 3A).

We next analyzed the molecular mechanism responsible for Lrrk2-stimulated NF-κB transcriptional activity. Interestingly, degradation of IκB protein, a prerequisite event for nuclear translocation of NF-κB, was not different in control and Lrrk2-KD cells; in cells of both clonal types, IκB protein was decreased at approximately 30 min, but rebounded 60 min after LPS activation (Fig. 3B). This result suggests that diminished NF-κB transcriptional activity in Lrrk2-KD cells is mediated by disruption of a molecular mechanism downstream of IκB degradation.

**Figure 4:**
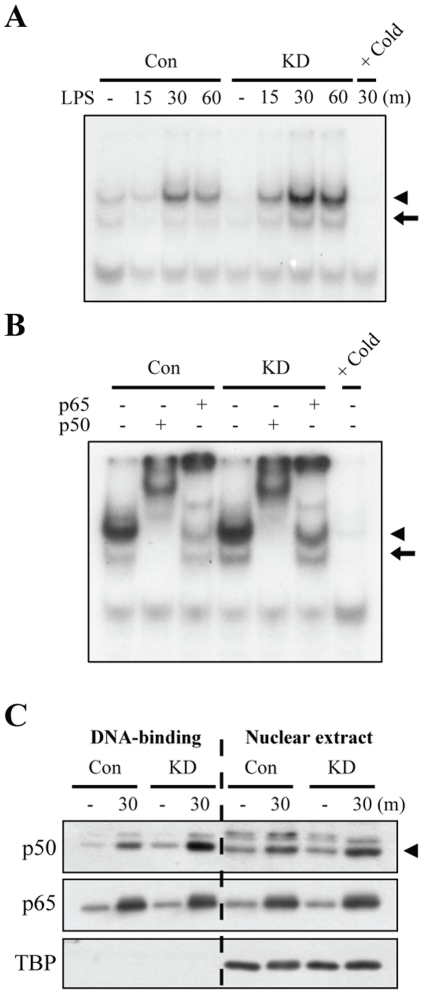
The DNA-binding ability of p50 is increased in Lrrk2-KD microglia in response to LPS stimulation. (**A**) The DNA-binding activity of NF-κB was analyzed by EMSA. Nuclear extracts were prepared from control and Lrrk2-KD cells at the indicated times after LPS treatment. Specific binding was analyzed using an excess (20×) of unlabeled (Cold) consensus NF-κB sequence. Two specific NF-κB-DNA complex bands (arrowhead and arrow) were detected. (**B**) A supershift assays were performed using NF-κB p50 and p65 antibodies. Nuclear extracts obtained 30 min after LPS treatment were preincubated with antibodies. Arrows and arrowheads in (A, B) indicate p50/p50 complex and p50/p65 complex, respectively. (**C**) DNA affinity precipitation assays were performed using nuclear extracts prepared from untreated and LPS-treated control and Lrrk2-KD microglia. Nuclear extracts (100 µg protein) were incubated with biotin-labeled NF-κB consensus sequence, and then precipitated with streptavidin-conjugated agarose beads. The amount of p50 or p65 in the nuclear extracts (nuclear extract), and bound to DNA (DNA-binding) were analyzed by Western blotting. TBP was used as a nuclear marker. The arrowhead in (C) indicates p50. Data are representative of three independent experiments.

A further investigation of the mechanism of Lrrk2 regulation of NF-κB activity using EMSA analyses unexpectedly showed that both lower (Fig. 4A, arrow) and upper (Fig. 4A, arrowhead) NF-κB-DNA complexes, identified in supershift assays as p50/p50 homodimers and mainly p50/p65 heterodimers, respectively (Fig. 4B), were somewhat more strongly detected in Lrrk2-KD cells than in control cells between 15 and 60 min after LPS stimulation. It has been reported that, unlike p50/p65 heterodimers, p50/p50 homodimers function to inhibit transcription [35]. Thus, we analyzed nuclear proteins that bound to the NF-κB binding DNA consensus sequence. For this, we incubated nuclear extracts with a 5′-biotinylated DNA probe and then precipitated with streptavidin-agarose resin. Within 30 min, LPS induced the nuclear translocation of p50 and p65 NF-κB subunits to a comparable degree in both Lrrk2-KD and control cells (Fig. 4C, Nuclear extract). Importantly, however, more p50 bound to DNA in Lrrk2-KD cells; p65 binding was not different in these two types of cells (Fig. 4C, DNA-binding). These results suggest that NF-κB transcriptional activity is reduced in Lrrk2-KD cells because the affinity of the inhibitory p50 subunit for the NF-κB consensus sequence is higher in these cells than in controls.

### Effect of overexpression of LRRK2-WT and LRRK2 mutants on LPS-induced inflammatory responses

**Figure 5:**
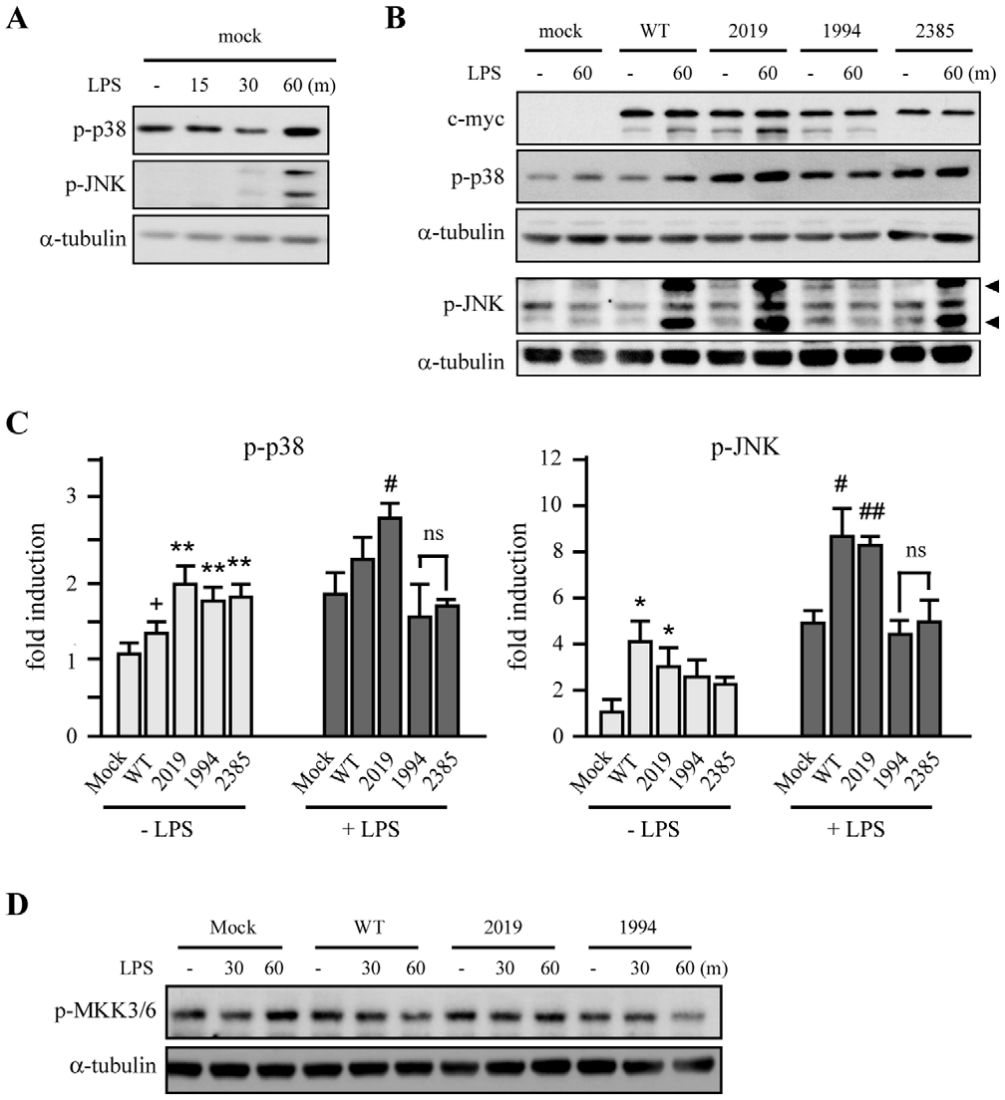
Effect of hLRRK2 overexpression on p38 and JNK phosphorylation in HEK293T cells. (**A**) LPS-responsive HEK293T cells (TCM-HEK), prepared as described in Materials and Methods, were co-transfected with empty pcDNA3.1 for the following control experiments. LPS (100 ng/mL) induced phosphorylation of p38, and JNK was as analyzed by Western blotting. (**B, D**) TCM-HEK cells co-transfected with c-myc-tagged hLRRK2 (WT, G2019S [2019], D1994A [1994], and G2385R [2385]) were treated with LPS (100 ng/mL) for the indicated times. Empty vector (mock) was used as a control. c-myc and α-tubulin were used as markers of LRRK2 expression and loading controls, respectively. Phosphorylation levels of p38, JNK, and MKK3/6 were analyzed by Western blotting. Phosphorylation of JNK was indicated with arrowhead. (**C**) Band intensities in (B) were quantified using a densitometer. Values are means ± SEMs of three independent experiments (*+p* = 0.054, *p<0.05, **p<0.01 vs. mock in LPS-untreated group [−LPS]; *#p*<0.05; ##*p<0*.01 vs. mock in LPS-treated group [+LPS]; *ns*, not significant). Data are representative of three independent experiments.

We next examined the effect of overexpression of hLRRK2 on LPS-induced activation of MAPKs and NF-κB using HEK293T cells. These cells were used because of low transfection efficiency of BV-2 microglia. Because parental HEK293T cells do not respond to LPS treatment [36], we transiently transfected HEK293T cells with expression plasmids for the LPS receptor, TLR4, and proteins of the downstream signaling machinery, CD14 and MD2 [37], [38]. In these LPS-responsive TCM-HEK cells, LPS induced phosphorylation of p38 and JNK within 60 min (Fig. 5A). Thus, we analyzed the effect of LRRK2-WT and LRRK2 mutants on basal and LPS-induced p38 and JNK phosphorylation levels in TCM-HEK cells cotransfected with LRRK2-expression vectors or empty vector (mock). Overexpression of LRRK2-WT and all LRRK2 mutants, including kinase-active (G2019S), kinase-dead (D1994A), and WD-domain (G2385R) mutants, tended to increase basal levels of phosphorylated p38 compared to empty vector-transfected (mock) cells, although the extent of the increase varied depending on the specific LRRK2 variant. Basal levels of phosphorylated JNK were also increased by overexpression of LRRK2-WT and LRRK2-G2019S, but not by overexpression of LRRK2-D1994A or LRRK2-G2385R (Fig. 5B, 5C). LPS increased p38 and JNK phosphorylation in cells transfected with LRRK2-WT, LRRK2-G2019S, and in mock-transfected cells, but not in cells transfected with LRRK2-D1994A or LRRK2-G2385R (Fig. 5B, 5C). Notably, LPS-induced p38 phosphorylation was significantly increased in LRRK2-G2019S-expressing cells, and LPS-induced JNK phosphorylation was increased in LRRK2-WT and LRRK2-G2019S-expressing cells above the levels in mock cells (Fig. 5B, 5C). The kinase-active mutant G2019S, in particular, enhanced both basal and LPS-induced levels of phosphorylated (activated) p38 and JNK, whereas the effects of other LRRK2 mutants on p38 and JNK activation were variable. However, as in microglia, overexpression of LRRK2-WT, -G2019S, and -D1994A had no effects on p-MKK3/6 levels in TCM-HEK cells in the absence and presence of LPS (Fig. 5D).

**Figure 6:**
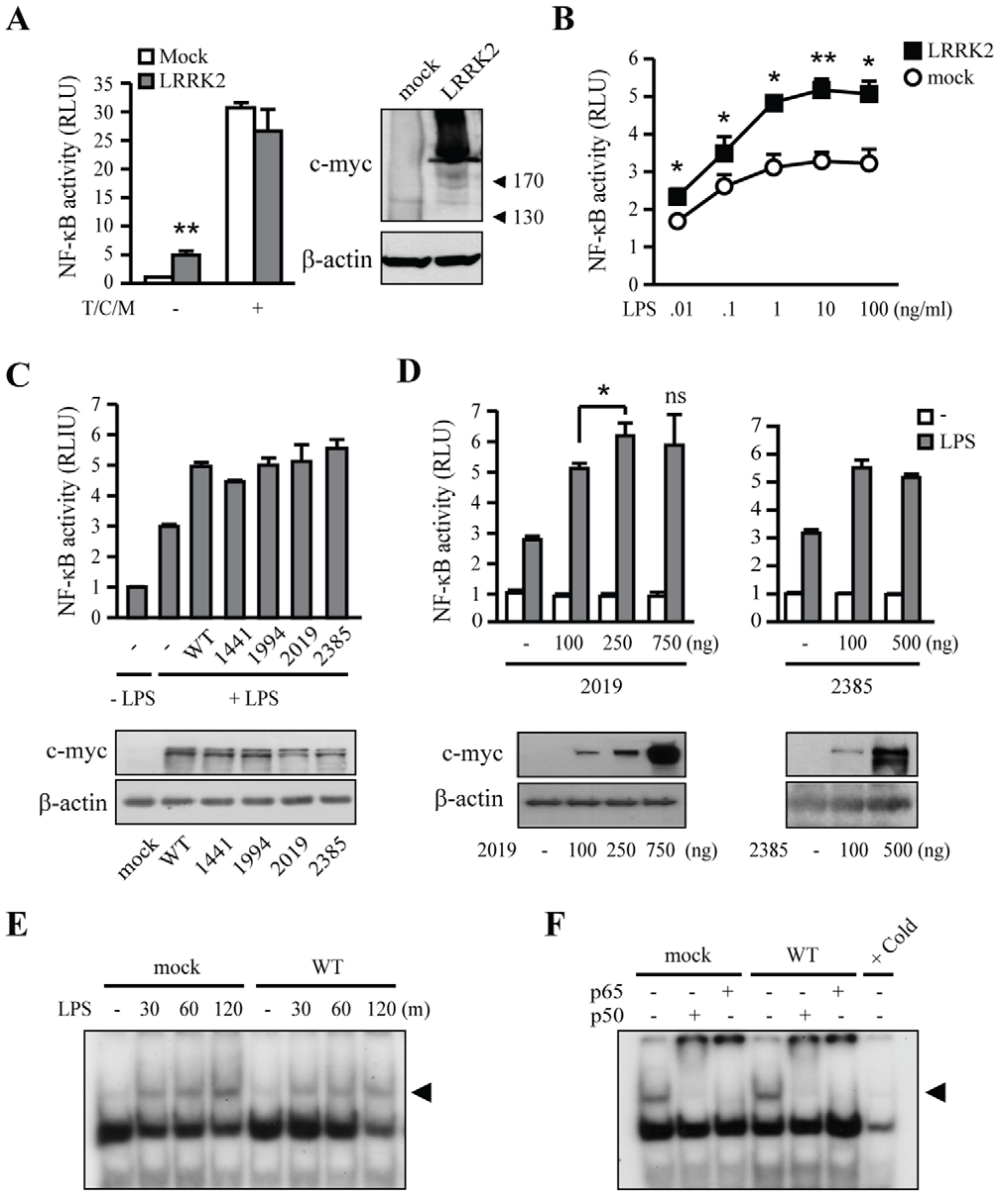
Effect of hLRRK2 overexpression on the NF-κB signaling pathway and NF-κB activity in HEK293T cells. (**A**) HEK293T cells (parental or TCM-HEK) were transfected with a 5× NF-κB–luciferase reporter construct, empty vector (mock), or hLRRK2 expression vector. One unit is the basal NF-κB activity detected in untreated parental HEK293T cells. *Left:* NF-κB activity was measured by luciferase assay. *Right:* LRRK2 expression was confirmed by Western blotting for c-myc. β-actin was used as a loading control. Values are means ± SEMs of three independent experiments. (**p<0.01 vs. mock). (**B**) LPS induced a dose-dependent increase in NF-κB activity in TCM-HEK cells with or without LRRK2. Cells were treated with the indicated amount of LPS for 6 h. The values shown are fold-induction relative to the values of NF-κB activity in unstimulated TCM-HEK cells, expressed as means ± SEMs of three independent experiments (*p<0.05, **p<0.01 vs. mock). (**C**) *Upper panel:* TCM-HEK cells were transfected with LRRK2-WT and each LRRK2 mutant, and NF-κB activity was analyzed after treating with LPS (10 ng/mL) for 6 h. *Lower panel:* Expression of LRRK2 mutants was detected by Western blotting. Values are means ± SEMs of three independent experiments. (**D**) TCM-HEK cells were transfected with mock, G2019S (100, 250 and 750 ng), and G2385R (100 and 500 ng) hLRRK2 expression vector. Values are means ± SEMs of three independent experiments. *Upper panel:* NF-κB activity measured by luciferase assay. *Lower panel:* LRRK2 levels determined by Western blotting (*p<0.05; *ns*, not significant). (**E**) NF-κB DNA-binding activity was analyzed by EMSA. Nuclear extracts were obtained from mock and LRRK2-WT-overexpressing TCM-HEK cells stimulated with LPS (10 ng/mL) for the indicated times. (**F**) A supershift assay was performed using nuclear extracts obtained 1 h after LPS treatments and preincubated with NF-κB p50 and p65 antibodies. Arrowheads indicate p50/p65 complex. Specific binding (arrowhead) was analyzed using an excess (20×) of unlabeled (Cold) DNA.

Next, we examined whether overexpression of LRRK2-WT or LRRK2 mutants modified NF-κB transcriptional activity. Expression of LRRK2-WT increased NF-κB transcriptional activity in HEK cells (left panel in Fig. 6A) as previously reported [16]. However, expression of TCM alone significantly enhanced NF-κB transcriptional activity in HEK cells (Fig. 6A), and co-expression of LRRK2-WT did not further increase NF-κB transcriptional activity (left panel in Fig. 6A). Expression of LRRK2 was confirmed with Western blot (right panel in Fig. 6A). Next, we evaluated the effect of overexpression of LRRK2-WT on LPS-induced NF-κB transcriptional activity in TCM-HEK cells: LRRK2-WT compared to empty vector enhanced LPS-induced NF-κB activity (Fig. 6B). This increase in NF-κB activity was LPS-dose dependent in the range of 0.01–100 ng/mL, with maximum activity obtained with 1 ng/mL LPS (Fig. 6B). We further analyzed the effect of other LRRK2 mutants on NF-κB activity. Interestingly, all LRRK2 mutants (R1441C, G2019S, G2385R, and D1994A) significantly increased NF-κB activity, indicating that the increase was independent of mutations in ROC, kinase, and WD domains (Fig. 6C). In addition, G2019S showed dose-response at 100 and 250 ng, but not at 750 ng (Fig. 6D). G2385R also similarly increased NF-κB activity at 100 ng and 500 ng (Fig. 6D). Thus, the effect of Lrrk2 on NF-κB activity may be saturated at quite low Lrrk2 levels.

Next, we further analyzed the effect of LRRK2 overexpression on the formation of NF-κB-DNA complexes in TCM-HEK cells since the p50 binding to the NF-κB consensus sequence increased in response to LPS in Lrrk2-KD microglia (Figs. 4 and 6E–F). However, there was no significant difference in NF-κB-DNA complex formation in LRRK2-overexpressing and vector-transfected (mock) cells between 30 min and 2 h after LPS activation (Fig. 6E, arrowhead). Supershift assay with p50 and p65 antibodies showed that the NF-κB-DNA complex is p50/p65 heterodimers, and no p50/p50 homodimer was detected in TCM-HEK cells (Fig. 6F).

Taken together, the results of this study suggest that LRRK2 regulates inflammation in settings as diverse as murine microglia and TCM-HEK cells. Furthermore, p38 and JNK MAPK, and NF-κB signaling pathways are possible targets of LRRK2.

## Discussion

Although PD is caused by the degeneration of dopaminergic neurons in the SN, the cause of neuronal loss could reflect changes in the brain environment as well defects in dopaminergic neurons. Several studies have reported that PD-related genes regulate glial function. Mitochondrial damage is detected in astrocytes, microglia, and oligodendrocytes in the SN of parkin (Park2) knockout (KO) mice and mutant α-synuclein-expressing mice [39]. Astrocytes prepared from DJ-1 (Park7) KO mice produce higher levels of inflammatory mediators in response to LPS than astrocytes prepared from control mice [40]. Mixed glial preparations from parkin KO mice are more susceptible to 1-methyl-4-phenylpyridine (MPTP)- and epoxomicin-induced cell death compared to mixed glia from wild-type mice [41], [42]. The results of the current study add to these observations, providing evidence that abnormalities of LRRK2 alter inflammation, which are considered risk factors for PD.

MAPK subtypes p38, JNK, and ERK are important signaling molecules that mediate microglial inflammatory activation induced by several stimulators, including LPS, gangliosides, and thrombin [31]–[33]. We found that p38 and JNK, but not ERK, appeared to be linked to LRRK2 in LPS signaling. In Lrrk2-KD microglia, p38 activation decreased whereas JNK and ERK were unaffected (Fig. 2). And in TCM-HEK cells, overexpression of LRRK2-WT and various LRRK2 mutants altered the phosphorylation levels of JNK and p38 (Fig. 5). LRRK2 was previously reported to phosphorylate MKK3/6 and MKK7, upstream kinases of p38 and JNK, in HEK293T cells [43], [44]. Unexpectedly, however, basal and LPS-induced MKK3/6 phosphorylation levels were not changed in Lrrk2-KD microglia and hLRRK2-overexpressing TCM-HEK cells compared to that in parental cells (Figs. 2D, 2E and 5D). Therefore, LRRK2 may regulate p38 through MKK-independent pathway(s) in microglia and TCM-HEK cells. Accordingly, in the immune system, TAK1-binding protein 1 (TAB1) has been suggested as a new upstream regulator of p38 [45]. We also speculate that TCM expression may alter cellular characteristics of HEK293T cells. Thus LRRK2 overexpression may activate p38 MAPK through MKK3/6-independent pathway(s) in TCM-HEK.

Although ERK activation is involved in LRRK2-G2019S-induced cell death and neurite shortening [23], [46], [47], it was difficult to reach a conclusion about the effect of LRRK2 overexpression on ERK activation in TCM-HEK cells, because LPS barely activated ERK and the basal levels of ERK activation exhibited too much variation between experiments. The enhanced kinase activity of G2019S, which is the most prominent LRRK2 mutant found in PD patients, has been suggested to be crucial for the neurotoxicity of this mutant [5], [6], [23]. However, whether the kinase activity of LRRK2 is linked to the regulation of inflammatory responses is questionable. TCM-HEK cells that expressed LRRK2-WT and/or LRRK2-G2019S similarly enhanced both basal and LPS-induced phosphorylation of p38 and JNK (Fig. 5B, 5C), whereas only basal p38 activity was increased in cells that expressed LRRK2-D1994A (a kinase dead mutant), and these cells lost the ability to react to LPS (Fig. 5B, 5C). Therefore, the kinase activity of LRRK2 appeared to be important for enhancing LPS-induced inflammatory responses. The WD-domain mutant, LRRK2-G2385R, similar to LRRK2-D1994A, regulated p38 and JNK activation in the absence and presence of LPS, although the kinase activity of LRRK2-G2385R was not directly analyzed. Additionally, we found that IFN-γ-induced iNOS expression and NO production were significantly reduced in Lrrk2-KD microglia (Figure S2A, S2B). Furthermore, IFN-γ-induced phosphorylation of p38 and JNK but not phosphorylation of STAT-1 was reduced in Lrrk2-KD microglia (Figure S2C, S2D). In agreement with our results, leukocytes and PMBCs isolated from PD patients carrying the LRRK2-G2019S mutant exhibited differential expression and activation of p38 and JNK, but not ERK, compared to healthy controls [19], [48].

Activation of the transcription factor NF-κB is essential for the expression of inflammatory mediators in microglia [32]–[34]. We observed that LPS-induced transcriptional activity of NF-κB was significantly inhibited in Lrrk2-KD microglia (Fig. 3A). However, the mechanisms underlying LRRK2 regulation of NF-κB transcriptional activity may not be simple. Several studies have reported that p38 MAPK enhances NF-κB transcriptional activity [49]–[51]. Accordingly, we examined whether decreased NF-κB transcriptional activity in Lrrk2-KD cells was related to decreased p38 MAPK activity. However, both pharmacological inhibition of p38 with SB203580 and siRNA-mediated p38 knockdown enhanced rather than inhibited NF-κB luciferase activity in microglia (data not shown), suggesting that Lrrk2 affects NF-κB transcriptional activity independently of p38 MAPK. Neither degradation of IκB nor nuclear translocation of NF-κB proteins, p65 and p50, were different between control and Lrrk2-KD microglia (Figs. 3B, 4C). However, EMSA supershift assays and DNA affinity precipitation experiments showed that Lrrk2 regulated NF-κB-DNA complex formation; particularly, the amount of p50/p50 homodimer-DNA complexes, which have been suggested to act as an inhibitory transcription factor, was increased in Lrrk2-KD microglia compared to control cells (Fig. 4A–C). As an effort to reveal how p50/p50 homodimer-DNA complexes increased in Lrrk2-KD cells, we examined phosphorylation of serine 337 (p-Ser337) of p50, which is important for DNA binding of p50/p50 homodimer [52]. However, the p-Ser337 levels were not altered in Lrrk2-KD cells with or without LPS treatment (data not shown). We also examined phosphorylation of serine 276 (p-Ser276), and serine 536 (p-Ser536) of p65 since posttranslational modification of p65 is important for NF-κB transcriptional activity independently of NF-κB subunit dimerization and/or IκB-mediated nuclear translocation [53]–[55]. However, we found no significant changes in p-Ser276 or p-Ser536 levels of p65 in Lrrk2-KD microglia (data not shown). Therefore, additional studies are required to reveal how LRRK2 regulates NF-κB activity.

Increasing attention has gradually focused on the importance of neuroinflammation as a risk factor in the pathogenesis of PD [12], [13]. For example, genetic variations in the inflammation-regulatory gene, HLA, were found to be associated with late-onset, sporadic PD [56], which is in an accord with previous finding that HLA-DR-positive microglia are commonly found in the SN of PD patients [57]. Although PD mouse models carrying hLRRK2 pathogenic mutants fail to develop nigrostriatal neurodegeneration despite the presence of age-dependent motor deficits [7]–[10], animal models in which viruses carrying hLRRK2 pathogenic mutants are directly injected into the striatum do develop nigral neurodegeneration of infected neurons [58], [59]. Direct injection of virus could induce inflammatory responses, which cooperate with pathogenic LRRK2 to develop nigrostriatal neurodegeneration. Previous reports have shown that persistent systemic inflammation induces the loss of dopaminergic neurons in parkin-deficient mice [60], supporting the hypothesis that inflammation enhances the toxicity of genetic factors and/or environmental toxins. In addition, central and systemic IL-1 expression in an animal model was shown to enhance 6-hydroxydopamine-mediated neurodegeneration in the SN [61].

The results of this study indicate that LRRK2 is a positive regulator of brain inflammation. Therefore, in injury states in which inflammation occur, LRRK2 abnormally regulates brain inflammation, which could cooperate with neuronal defects produced by abnormal LRRK2 and further increase the risk of developing PD.

## Supporting Information

Figure S1 **LRRK loss-of-function mutant express low levels of antimicrobial proteins (AMP) genes.** Wild type (w^1118^) and LRRK loss-of-function mutant (LRRK^P1^) fly were a gift from Dr. Chung in Seoul National University, Korea [30]. (**A**) Expression levels of drosophila *Rpl32*, *attacin A*, *diptericin* and *LRRK* were analyzed by conventional RT-PCR. The following primers were used for amplification of the target genes: *Rpl32*, 5′-AGATCGTGAAGAAGCGCACCAAG-3′ (sense) and 5′-CACCAGGAACTTCTTGAATCCGG-3′ (antisense); *Attcin A*, 5′-ACAAGCATCCT AATCGTGGC-3′ (sense) and 5′-TCAGATCCAAACGAGCATCAG-3′ (antisense); *Diptericin*, 5′-TTTGGCTTATCCGATGCCCG-3′ (sense) and 5′-ATGGTCCTCCCAAGTGCTGT-3 (antisense); *LRRK*, 5′-GTGGCTGTCGGAACGCATAAC-3′ (sense) and 5′-GCCGCACCACAATTCATAG-3′ (antisense). (**B**) mRNA levels of AMPs, attacin A and diptericin, were quantified by qRT-PCR at 5 or 30 day after eclosion. mRNA was prepared from a total of 25–30 flies. The mRNA level of Rpl32 was used as an internal control. Values are means ± SEM of three independent experiments (**p<0.01 vs. control fly).
(TIF)

Figure S2 **IFN-γ-induced inflammatory responses are attenuated in Lrrk2-KD microglia.** Control (Con) and Lrrk2-KD (KD) cells were treated with 10 ng/ml mouse IFN-γ for the indicated times (A, C, D) and 48 h (B). (**A, C, D**) Levels of iNOS protein (A), phospho-STAT1 (Tyr701) (C) and phospho-p38 and –JNK (D) were analyzed by Western blotting. α-tubulin was used as an internal control. Data are representative of three independent experiments. (**B**) The amount of nitrite converted from NO in the media was measured using Griess reagent as described in methods. Values are means ± SEM of three samples. *, p<0.05 vs. control.
(TIF)
