# Supplementary figures and images for: Impaired Inflammatory Responses in Murine Lrrk2-Knockdown Brain Microglia
Source: PLoS One. 2012 Apr 9;7(4):e34693. doi: 10.1371/journal.pone.0034693 (PMC3322140; doi:10.1371/journal.pone.0034693)

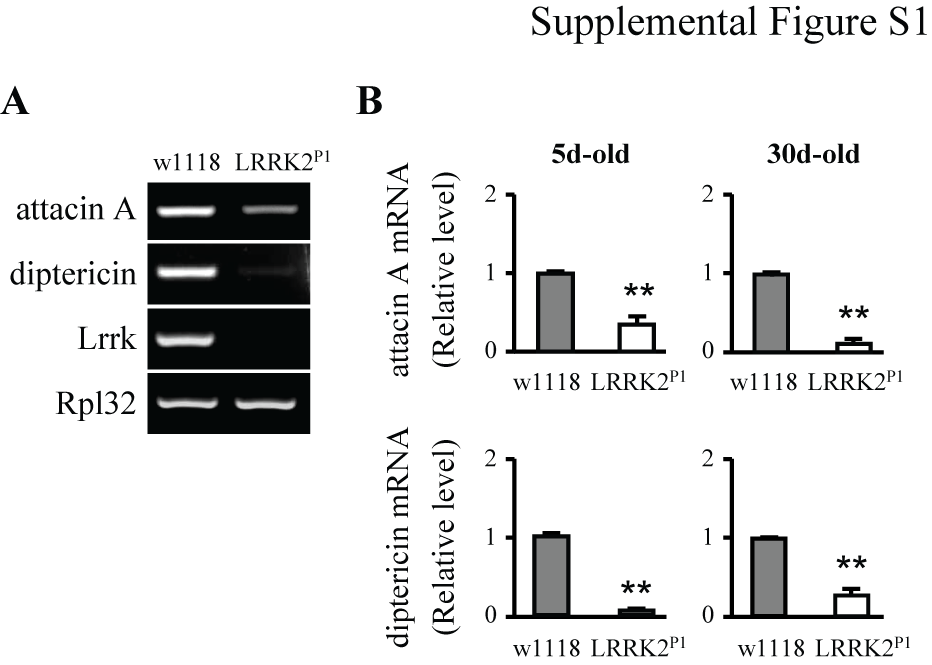

Supplement: Figure S1 — LRRK loss-of-function mutant express low levels of antimicrobial proteins (AMP) genes. Wild type (w1118) and LRRK loss-of-function mutant (LRRKP1) fly were a gift from Dr. Chung in Seoul National University, Korea [30]. (A) Expression levels of drosophila Rpl32, attacin A, diptericin and LRRK were analyzed by conventional RT-PCR. The following primers were used for amplification of the target genes: Rpl32, 5′-AGATCGTGAAGAAGCGCACCAAG-3′ (sense) and 5′-CACCAGGAACTTCTTGAATCCGG-3′ (antisense); Attcin A, 5′-ACAAGCATCCT AATCGTGGC-3′ (sense) and 5′-TCAGATCCAAACGAGCATCAG-3′ (antisense); Diptericin, 5′-TTTGGCTTATCCGATGCCCG-3′ (sense) and 5′-ATGGTCCTCCCAAGTGCTGT-3 (antisense); LRRK, 5′-GTGGCTGTCGGAACGCATAAC-3′ (sense) and 5′-GCCGCACCACAATTCATAG-3′ (antisense). (B) mRNA levels of AMPs, attacin A and diptericin, were quantified by qRT-PCR at 5 or 30 day after eclosion. mRNA was prepared from a total of 25–30 flies. The mRNA level of Rpl32 was used as an internal control. Values are means ± SEM of three independent experiments (**p<0.01 vs. control fly). (TIF) [file pone.0034693.s001.tif]

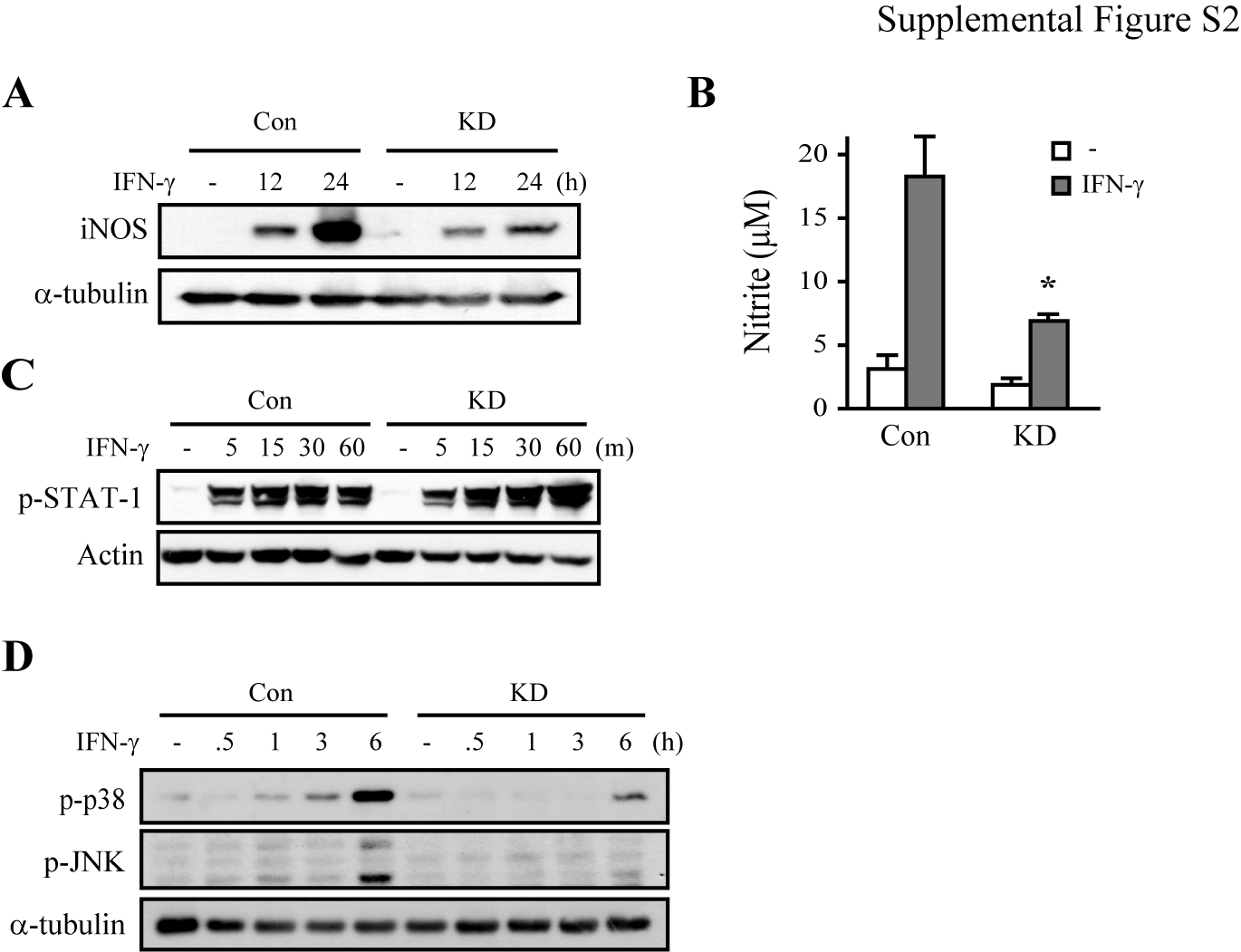

Supplement: Figure S2 — IFN-γ-induced inflammatory responses are attenuated in Lrrk2-KD microglia. Control (Con) and Lrrk2-KD (KD) cells were treated with 10 ng/ml mouse IFN-γ for the indicated times (A, C, D) and 48 h (B). (A, C, D) Levels of iNOS protein (A), phospho-STAT1 (Tyr701) (C) and phospho-p38 and –JNK (D) were analyzed by Western blotting. α-tubulin was used as an internal control. Data are representative of three independent experiments. (B) The amount of nitrite converted from NO in the media was measured using Griess reagent as described in methods. Values are means ± SEM of three samples. *, p<0.05 vs. control. (TIF) [file pone.0034693.s002.tif]
